# Are There Sex Differences in How Social Cohesion and Loneliness Relate to Cognitive Decline in Latinos?

**DOI:** 10.1093/geroni/igaf122.1673

**Published:** 2025-12-31

**Authors:** Graciela Muniz-Terrera, Alejandra Marroig, Angela Gutierrez, Courtney Thomas Tobin, Barış Sevi

**Affiliations:** Ohio University, Athens, Ohio, United States; Universidad de la Republica Uruguay, Montevideo, Montevideo, Uruguay; Western University of Health Sciences, Pomona, California, United States; University of California, Los Angeles Fielding School of Public Health, Los Angeles, California, United States; MEF University, Maslak, Istanbul, Turkey

## Abstract

Latinos are the largest minoritized population in the US, and therefore, understanding cognitive decline in this population is paramount. Previous literature has identified exogenous factors, such as social cohesion and endogenous factors, such as loneliness, as associated with cognitive decline in older men and women. To improve our understanding of the role of social cohesion and loneliness on cognitive decline in Latino older adults, we fitted independent linear mixed effects models to cognitive scores from men and women aged 50 and older (n = 2,321) who participated in the Health and Retirement Study (2006 - 2016), accounting for both, social cohesion and loneliness. Models were also adjusted for sociodemographic factors. In men and women, social cohesion was positively associated with baseline cognitive function (p < 0.001), while loneliness was negatively associated with baseline cognitive function (p < 0.001). The effect size of loneliness on baseline cognition in men was 3-fold the effect of social cohesion in men, while in women, it was 2-fold. However, none of these factors were significantly associated with cognitive trajectories over time. These findings highlight the importance of the role of exogenous and endogenous domains of the exposome in cognitive function among Latino adults. Health promotion initiatives should focus on implementing culturally appropriate strategies that enhance social cohesion within neighborhoods and help reduce feelings of loneliness.

